# Utility of Indigenously Developed Square Grid Method for Evaluation of Tumor-Stroma Ratio and Stromal Tumor-Infiltrating Lymphocytes in Invasive Breast Carcinoma: A Pilot Study

**DOI:** 10.30699/IJP.2023.1989528.3063

**Published:** 2023-07-16

**Authors:** B. N. Kumarguru, A. S. Ramaswamy, C. A. Arathi, D. Swathi

**Affiliations:** Department of Pathology, PES Institute of Medical Sciences and Research, Kuppam, Chittoor, Andhra Pradesh, India

**Keywords:** Cancer, Inter-observer variability, Pathology, Technique

## Abstract

**Background & Objective:**

Invasive breast carcinoma (IBC) is the most commonly diagnosed cancer among women in India. The conventional visual method of evaluation of Tumor-Stroma Ratio (TSR) and Stromal Tumor-Infiltrating Lymphocytes (sTIL) appears to be subjective. The present study aims to evaluate the utility of the indigenously designed square grid method for the evaluation of tumor-stroma ratio and stromal tumor-infiltrating lymphocytes in invasive breast carcinoma by assessing the inter-observer variability.

**Methods:**

This was a retrospective study conducted at a rural tertiary care referral institute from July 2018 to June 2020. In each case, microphotographs were taken from 10 representative fields in H&E-stained sections for evaluating TSR in low-power and sTIL in high-power. Both the parameters were evaluated employing an indigenously designed square grid applied onto microphotographs in the power-point slides by making use of principles of the Pythagorean theorem. Both parameters were separately evaluated by two pathologists. Cohen kappa statistics was the statistical tool used to analyze inter-observer variability.

**Results:**

Thirty cases were analyzed. Invasive breast carcinoma of no special type (IBC-NST) was the most common histopathological type (26 cases (86.67%)). For TRS evaluation, a Kappa value of 0.78 suggested substantial agreement with an agreement of 91.67%. For sTIL evaluation, a Kappa value of 0.51 suggested moderate agreement with an agreement of 88.33%. The P-values were statistically highly significant (*P*<0.001).

**Conclusion:**

Square grid method is a novel technique for evaluating TSR and sTIL in invasive breast carcinoma. It can be considered an example of the application of Pythagoras’ theorem in Pathology.

## Introduction

Invasive breast carcinoma (IBC) is the most commonly diagnosed cancer among women in India, attributed to the advent of population growth, lifestyle modifications, and migration from rural to urban areas (1). It is considered the leading cause of cancer-related deaths in the Western world (2). IBC is a heterogeneous disease. Hence distinguishing patients who need more aggressive therapy from patients who would benefit from a conservative approach remains a tough challenge. 

The interaction between the tumor cells and the cells in the tumor microenvironment has gained significant interest in the last two decades (3). Tumor-stroma ratio (TSR) is a parameter that translates the amount of tumor-associated stroma (4). It has been shown to be of high value in breast cancer, colon cancer, gastric cancer, and esophageal cancer (3). Tumor-infiltrating lymphocytes (TIL) are considered to reflect the host’s immune response against cancer (5). 

TSR and TIL are the parameters of paramount research interest in invasive breast carcinoma in recent years. The conventional visual method of evaluation of TSR and the TIL appears to be subjective. The use of sophisticated software may be expensive and may not be feasible in all laboratories. The present study makes use of the Pythagorean theorem to indigenously design a square grid for the evaluation of the Tumor-Stroma Ratio (TSR) and Stromal Tumor Infiltrating Lymphocytes (sTIL). 

Pythagorean theorem was known earlier in India and China even before the sixth century. Baudhayana, an Indian mathematician discussed the theorem around 800 BC in his book Baudhayana-Sulba-Sutra (6). Pythagoras’ theorem is the fundamental law of geometry describing the relationships of three sides of a right-angled triangle (7). It has found many applications in different fields, particularly the engineering field (6). Its application in pathology needs to be explored. The present study is a novel one in that it can be considered as an example of the application of Pythagoras theorem in pathology used for the purpose of analysis of microphotographs to evaluate histopathological parameters in IBC.

The present study is undertaken to assess the utility of the indigenously designed square grid method for the evaluation of tumor-stroma ratio and stromal tumor-infiltrating lymphocytes in invasive breast carcinoma by assessing the inter-observer variability. The focus of the study was to develop and standardize a cost-effective tool to evaluate TSR and stromal TIL. To the best of the authors’ knowledge, this appears to be a unique study wherein an indigenously designed square grid has been employed to evaluate histopathological parameters like tumor-stromal ratio and stromal tumor-infiltrating lymphocytes on the basis of Pythagoras theorem. 

## Methods and Materials

The study was conducted in the histopathology section of the Department of Pathology at a tertiary care referral Institute. It was an observational retrospective study of invasive breast cancer cases from July 2018 to June 2020 for a period of two years. It was a pilot study for the purpose of application of the concept in future studies. Histopathology slides of IBC slides were retrieved. The histological characteristics of IBCs were reviewed. IBCs were classified according to the WHO Classification of breast tumors, 5th edition [2019] (8). The study was approved by the Institutional ethics committee. All invasive breast carcinoma specimens received for histopathological evaluation were included in the study. Core biopsy specimens, lumpectomy specimens, and those breast tissue specimens that were unsatisfactory for evaluation were excluded from the study. 

Tumor-stroma ratio was assessed in H&E-stained sections in 10 low-power fields (X100) and documented. TSR was assessed according to a modified protocol, originally described by Mesker WE* et al.* (9). Stromal tumor-infiltrating lymphocytes were assessed in H&E-stained sections in 10 high-power fields (X400) and documented. Each high-power field corresponds with an area of 0.196 mm2. Stromal tumor-infiltrating lymphocytes were assessed according to the international working group 2014 guidelines with minor modifications to suit the protocol of the present study (10). Microphotographs were employed for better assessment and for the purpose of reproducibility.

The practical tools employed in the present study were microphotographs taken from a mobile camera, an indigenously designed square grid, and Microsoft Office power-point presentation document.

An indigenously designed square grid (KRAS square grid) consisting of 100 small squares of equal dimensions) was employed to estimate the values. The square grid was named after the authors involved in designing the methodology. The square grid was superimposed on microscopic images in a power-point slide. The dimensions of the square were calculated by applying Pythagoras’ theorem. The number of tumor-predominant squares, tumor-stroma-predominant squares, and lymphocyte-predominant squares in the tumor stroma were documented. TSR and sTIL were subsequently calculated. Both the parameters were separately evaluated by two pathologists and inter-observer variability was analyzed.

All microphotographs are taken from the same mobile camera (13 MP) to ensure that the image circle has the same diameter. The original microphotograph (without cropping) was pasted on the blank office theme of the power-point presentation document thereby ensuring that the original image magnification is not altered.

**Protocols:**

**Selection of Area for Microphotographs**

1. The invasive edge of invasive breast carcinoma was identified in the scanner (X4) objective.
2. The tumor stroma was screened in the tumor area at the invasive edge using a low-power (10X) objective.
3. Area was selected such that the tumor stroma was bound by the tumor on all sides of the image field.

**Taking Microphotographs for Tumor-Stroma Ratio Estimation**

1. Care was taken to exclude the areas corresponding to the stroma adjacent to the tumor (non-tumor stroma), necrotic areas, and mucinous areas.
2. The tumor stroma was evaluated in 10 random low-power fields (10X objective).
3. Microphotographs were taken from the representative areas.
4. Tumor percentage and stromal percentage were estimated by applying KRAS square grid over the microphotographs in power-point slides.

**Taking Microphotographs for Stromal Tumor-infiltrating Lymphocytes**

1. Care was taken to exclude the areas corresponding to the stroma adjacent to the tumor (non-tumor stroma), necrotic areas, hyalinized areas, crush artifacts, DCIS area, tertiary lymphoid structures, and mucinous areas.
2. Areas corresponding to sTIL were screened in high-power fields (40X objective).
3. The nature of inflammatory infiltrate was determined.
4. Areas corresponding to the infiltration of granulocytes were excluded.
5. Areas corresponding to mononuclear cells like lymphocytes and plasma cells were considered for evaluation.
6. Stromal TIL was evaluated in tumor stroma in 10 random high-power fields. The hotspots method of evaluation was avoided.
7. Microphotographs of the representative areas were taken for estimating sTIL in tumor stroma.
8. Stromal TIL was evaluated only in the tumor stroma and not within the tumor islands
9. Percentage of sTIL was estimated by applying KRAS square grid over the microphotographs.

**Construction of KRAS square grid**

1. The square of dimension 11.7cm x11.7cm was designed in MS Word by employing the table properties.
2. One large square was composed of 100 small squares of equal dimensions.
3. Each small square corresponds to 1% of the large square.

**Preparing TSR-TIL Power-Point Slides**

1. The title page was designed so as to display preliminary information (case number, biopsy number, and TSR-primary tumor or sTIL-primary tumor).
2. A new slide was inserted with an office theme blank format.
3. A microphotograph of the representative field to be evaluated was inserted from the corresponding image file using the insert picture tool.
4. Square grid was superimposed over the microphotographic image by copying and pasting from MS Word or a previous slide.
5. The dimensions of the square were calculated by applying Pythagoras’ theorem. The dimension (length) of the square grid (corresponding to the length of the cord of the circle) to be inscribed in the circle was calculated by using the formula:

Formula: a = d/√2

Where:

a = dimension (length) of the square grid to be inscribed in the circle

d = diameter of the circle as measured in slide view (full-screen view) of power-point

Example:

d =16.6cm (standard diameter for the majority of microscopic photos, but may vary)

a =16.6/√2 = 11.73cm

Length of one side of square grid = 11.73cm

Length of each small square in the square grid = 1.17 (11.7 ÷ 10)

1. Square grid table was selected, and the dimension was adjusted according to the calculated value.
2. Layout option located in the top toolbar was selected.
3. The cell size and table size were adjusted as per the required dimension derived from the calculation.
4. Square grid table was moved above/below/right/left to inscribe and fit into the circle. Care was taken not to pull the table to adjust the size as the dimensions may vary and it may not be a perfect square of equal dimensions.
5. Four points of the square were either touching the circumference of the circle or points were just inside the circle but not outside the circle.
6. Square grid was employed for the estimation of study parameters in the power-point slide show mode.
7. Ten similar slides were prepared in each case for ten microscopic images for evaluating TSR and sTIL separately.

**Fig. 1:**
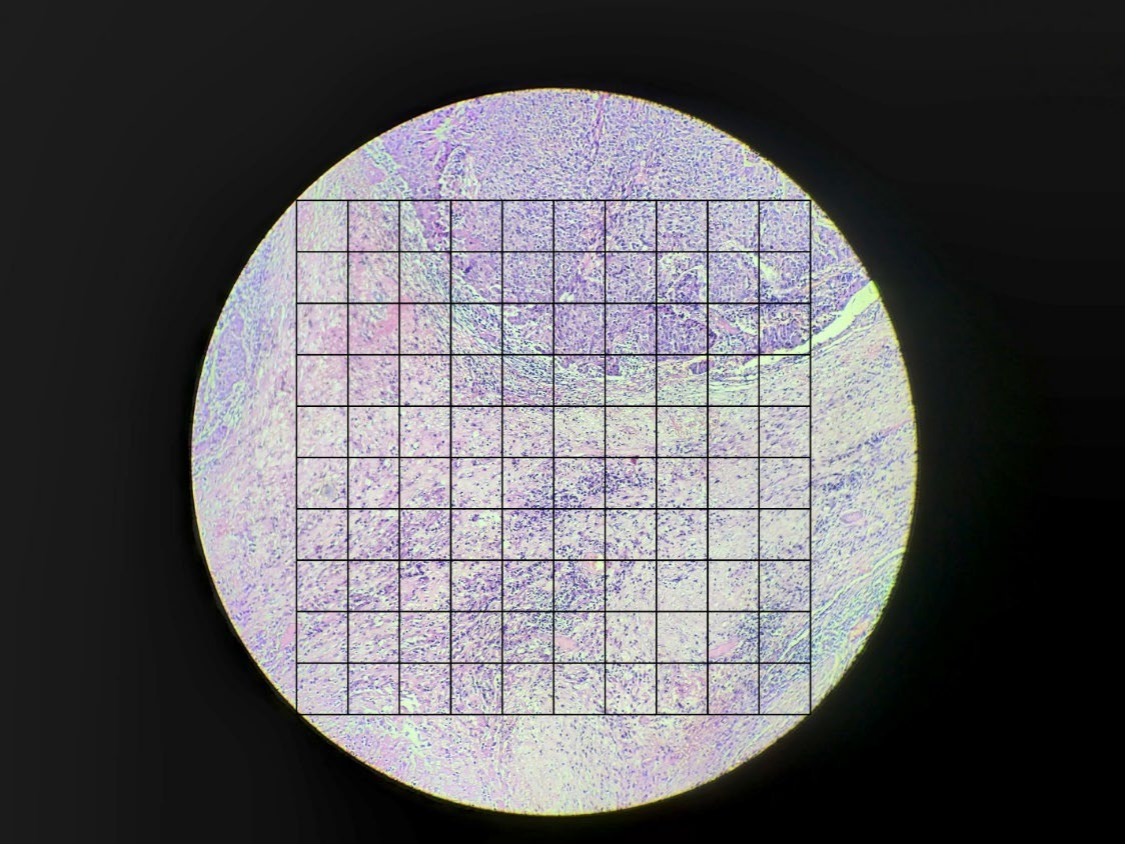
Superimposition of KRAS square grid over the microphotograph of invasive breast carcinoma for the evaluation of tumor-stroma ratio. (H&E, X100)

**Using KRAS Square Grid for Calculating Tumor-Stromal Ratio (** Figure 1 **)**

1. A number of small squares predominantly (approximately >50%) displaying the tumor were labeled as “T”, counted, and documented.
2. A number of small squares predominantly (approximately >50%) displaying the tumor stroma were labeled as “S”, counted, and documented.
3. A number of small squares that were inconclusive or had components of necrosis or empty space or artifacts were labeled as “NA”, counted, and documented.
4. TSR was calculated the following formula was used:

Tumor-stroma Ratio (%) = 

$\frac{\mathrm{Number of squares labeled as}"S"}{\mathrm{Number of labelled as}"T"+\mathrm{Number of squares lablledas}"S"}\times 100$

1. Reporting 0% and 100% in any given case was avoided.

**Fig. 2:**
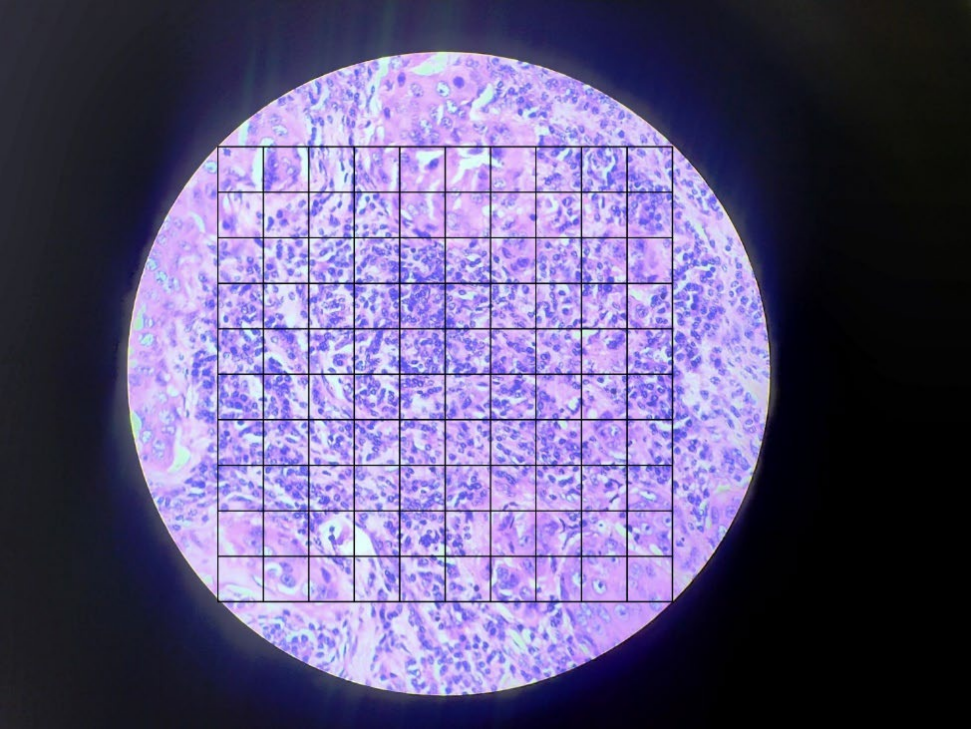
Superimposition of KRAS square grid over the microphotograph of invasive breast carcinoma for the evaluation of Stromal tumor-infiltrating lymphocytes (H&E, X400)

**Using KRAS Square Grid for Calculating Tumor-infiltrating Lymphocytes (** Figure 2 **)**

1. A number of small squares predominantly (approximately >50%) displaying the tumor stroma were labeled as “S”, counted, and documented.
2. Number of small squares predominantly (approximately >50% and/or ≥5 lymphocytes per small square) displaying the stromal tumor-infiltrating lymphocytes were labeled as “L”, counted, and documented.
3. If the image shows the presence of a tumor, the number of small squares predominantly (approximately >50%) displaying the tumor were labeled as “T”, counted, and documented only to exclude from calculation. This step may serve for verification purposes only.
4. Number of small squares that were inconclusive or had components of necrosis or empty space were labeled as “NA”, counted, and documented.
5. Stromal tumor-infiltrating lymphocytes percentage was calculated by using the formula:

Stromal tumor infiltrating lymphocytes (%) = $\frac{\mathrm{Number of squares labeled as}"L"}{\mathrm{Number of squares labeled as}"S"}\times 100$

**For Example 1 (Image showing the presence of tumor):**

If the number of squares predominantly displaying the carcinoma was 25, the number of squares displaying the tumor was 75 and the number of squares displaying predominantly displaying tumor-infiltrating lymphocytes in the tumor stroma was 50 

TIL = $\frac{50}{75}\times 100$** = **$\frac{5000}{75}$** = 66.67%**

**For Example 2 (Image showing no tumor):**

If the number of squares predominantly displaying the tumor was 0, the number of squares predominantly displaying stroma was 100 and the number of squares displaying predominantly displaying tumor-infiltrating lymphocytes in the tumor stroma was 50 

TIL = $\frac{50}{100}\times 100$** = **$\frac{5000}{100}$** = 50%**

1. Reporting 100% in any given case was avoided (because of the presence of empty spaces between lymphocytes).

**Scoring System**

A modified scoring system was employed to categorize the average percentages of TSR and sTIL separately in a given case. The scoring system categories were similar for TSR and TIL (Table 1). For the sake of feasibility and to avoid complexity, the decimal’s digits were rounded up to the nearest whole number. (Example: 67.76% was rounded up as 68%). Although the scoring system mentioned 0% and 100%, reporting 0% or 100% was considered logically not feasible in any given case. 

**Table 1:** The scoring system for Tumor-stroma ratio and Tumor-infiltrating lymphocytes

Scores	Tumor Stroma Ratio (%)	Tumor-infiltrating lymphocytes (%)
Score 0	0-25%	0-25%
Score 1	26-50%	26-50%
Score 2	51-75%	51-75%
Score 3	75-100%	75-100%

**Statistical Analysis of Data**

The socio-demographic variables and descriptive statistics were represented using frequencies and percentages. Inter-observer variability was analyzed by Cohen kappa statistics. Cohen suggested the Kappa results be interpreted as follows: value ≤ 0 as indicating no agreement, 0.01-0.20 as none to slight, 0.21-0.40 as fair, 0.41-0.60 as moderate, 0.61-0.80 as substantial and 0.81-1.00 as almost perfect (11). All results were analyzed by considering statistical significance at a level of P-value less than 0.05. All statistical analysis was performed with the statistical software STATA14.1. 

## Results

In the present study, 30 cases of invasive breast carcinoma were analyzed. The lesions were seen in only female patients in the age range of 36-70 years. Clustering of cases was seen in the sixth decade (Mean = 49.13 years; Median = 50 years). IBCs more commonly involved the left breast (16 cases (53.33%)) than the right breast (14 cases (46.67%)). The most common site of involvement was the upper-outer quadrant (15 cases (50%)) followed by the upper-inner quadrant (6 cases (20%)), central area (5 cases (16.67%), lower-outer quadrant (2 cases (6.67%)) and lower-inner quadrant (2 cases (6.67%)).

Invasive breast carcinoma of no special type (IBC-NST/Ductal carcinoma NST) was the most common histological type constituting 26 cases (86.67%) which included tumor-infiltrating lymphocyte (TIL) rich tumor (6 cases (20%)) and invasive breast carcinoma with neuroendocrine differentiation (1case (3.33%)), followed by invasive micropapillary carcinoma (3 cases (10%)), carcinoma with apocrine differentiation (1 case (3.33%)). Among the three cases of invasive micropapillary carcinoma, two cases (6.67%) were associated with IBC-NST, and one case (3.33%) was associated with mucinous carcinoma. (Table 2)

Lymph node metastasis was seen in 21 cases (70%). The lymphovascular invasion was identified in 16 cases (53.33%). Perineural invasion was identified in 2 cases (6.67%). The greatest dimension of the tumor ranged from 12 mm to 100 mm (mean = 41.43 mm). The most common primary tumor category was pT2 constituting 16 cases (53.33%). Common nodal categories were pN0 and pN1 constituting 9 cases (30%) each. The most common grade was grade 2 constituting 17 cases (56.67%). 

The agreement between the two pathologists (Pathologist 1 and Pathologist 2) with respect to TSR and sTIL was calculated by Cohen Kappa statistics. TSR ranged from 30.64% to 93.99% (Mean = 70.99%) (Figure 1). In the TRS assessment, score 4 was the most common score documented by both the Pathologists (Pathologist 1 and Pathologist 2). Pathologist 1 documented 14 cases (46.67%) underscore 4. Pathologist 2 documented 16 cases (53.33%) underscore 4. The mean score for TSR documented by Pathologist 1 was 3.3. The mean score for TSR documented by Pathologist 2 was 3.4. Kappa value (0.78) suggested substantial agreement for TRS evaluation with an agreement of 91.67%. The P-value was statistically highly significant (*P*<0.001). 

Stromal tumor-infiltrating lymphocytes ranged from 0% to 75.16% (Mean = 19.13%) (Figure 2). In the sTIL assessment, score 1 was the most common score documented by both pathologists. Pathologist-1 documented 22 cases (73.33%) underscore 1. Pathologist 2 documented 25 cases (83.33%) underscore 1. The mean score for sTIL documented by Pathologist 1 was 1.4. The mean score for sTIL documented by Pathologist 2 was 1.2. The Kappa value (0.51) suggested moderate agreement for sTIL evaluation with an agreement of 88.33%. The P-value was statistically highly significant (*P*<0.001) (Table 3).

**Table 2:** Distribution of the lesions of Invasive breast carcinoma

SL. No	Histopathology	Cases (n=30)
1	**Invasive Breast Carcinoma of no Specific type (IBC-NST)**	**26 (86.67%)**
	Invasive Breast Carcinoma of no Specific type (IBC-NST) – not associated with another specific subtype/ differentiating feature	19 (63.33%)
	TIL (Tumor-infiltrating lymphocytes) Rich IBC-NST	6 (20%)
	Invasive breast carcinoma with neuroendocrine differentiation	1 (3.33%)
2	**Invasive micropapillary carcinoma**	**3 (10%)**
	Associated with IBC-NST	2 (6.67%)
	Associated with mucinous carcinoma	1 (3.33%)
3	**Carcinoma with apocrine differentiation**	**1 (3.33%)**

**Table 3:** Interobserver reproducibility of TSR and TIL using KRAS square grid method in the Invasive breast carcinoma

Tumor-Stroma Ratio					
Parameters	**Pathologist 1**	**Pathologist 2**	**Kappa value**	**Agreement**	**P-value**
Score 1	0 (0%)	0 (0%)	0.78	91.67%	*P*<0.001 (Highly significant)
Score 2	5 (16.67%)	4 (13.33%)			
Score 3	11 (36.67%)	10 (33.33%)			
Score 4	14 (46.67%)	16 (53.33%)			
Average Score	3.3	3.4			
Tumor-Infiltrating Lymphocytes					
Score 1	22 (73.33%)	25 (83.33%)	0.51	88.33%	*P*<0.001 (Highly significant)
Score 2	4 (13.33%)	5 (16.67%)			
Score 3	4 (13.33%)	0 (0%)			
Score 4	0 (0%)	0 (0%)			
Average score	1.4	1.2			

## Discussion

Invasive breast cancer is the most common form of cancer in women and is a major cause of cancer-related death in women worldwide (12-16). The interaction between the tumor microenvironment and the tumor plays an important role in the progression of cancer, the metastatic potential of the tumor, and its resistance to chemotherapy (16). The tumor microenvironment has been found to be an important factor in the prognostication of epithelial tumors (17). The tumor stroma is composed of fibroblasts, bone-marrow-associated mesenchymal stem cells pericytes, adipocytes, macrophages, and immune cells. These components play an important role in neo-angiogenesis, tumor motility, tumor progression, and metastasis (18-20). The tumor stroma promotes tumor progression and metastasis by elaboration of various nutrients, chemokines, growth factors, and cytokines (14). 

The tumor-stroma ratio which is a part of the tumor microenvironment has been a topic of research interest (16). TSR is a promising parameter of prognostic value, which can be determined on routinely retrieved H&E-stained slides used for pathological assessment of surgically removed breast tissue (21). TSR is also an independent prognostic marker for IBCs (16). It is also a surrogate marker contributing to tumor aggressiveness (20). High TSR has been found to be associated with poor prognosis and adverse outcomes (20,22).

The role of immune system in the pathogenesis and clinical course of cancer is well established (23). But the role of immune response in breast cancer is not fully understood (15). Tumor-infiltrating lymphocytes (TIL) have been defined as mononuclear immune cells that infiltrate tumor tissue and constitute a continuous variable which is quantified as a percentage of area occupied by TIL per stromal area (12). TIL within the tumor and/or in the peritumoral sites have been recognized as an important biomarker that reflects the anti-tumor response in invasive breast cancer as well as other malignancies (24). The assessment of TIL within a tumor has been used as a surrogate measure of immune response (23). Tumor-Infiltrating lymphocytes are one of the best examples of the strict association that exists between natural defenses and carcinogenesis (25). Stromal tumor-infiltrating lymphocytes (sTIL) are associated with a patient’s immune response and offer a potentially favorable biological characteristic parameter (26). Stromal tumor-infiltrating lymphocytes represent a more reproducible parameter as they are frequently encountered and easier to detect and evaluate in H&E-stained sections (24). The onus for accurate estimation of stromal TIL falls on the pathologists (27). Stromal TILs have emerged as a potential prognostic and predictive marker in triple-negative breast cancer (TNBC) and HER2neu-positive cancers in the adjuvant and neoadjuvant setting (12,13,23,27). 

The practical tools employed in the present study were microphotographs taken from a mobile camera, an indigenously designed square grid, and a Microsoft Office power-point presentation document. The estimation of TSR and TIL is basically done by pathologists and values may be conveyed to the clinicians (oncologists and surgeons) to aid patient prognostication and further management.

In the present study, a square grid to be superimposed on microphotographs was indigenously designed based on Pythagoras theorem and was employed to evaluate histopathological parameters like tumor-stroma ratio and stromal tumor-infiltrating lymphocytes. The present study emphasizes the utility of this indigenously designed square grid method for estimating TSR and sTIL in invasive breast carcinoma in terms of inter-observer agreement. 

The number of cases was highest in the study conducted by Vangangelt KMH* et al.* (21). In contrast to the other studies, the present study had a lower number of cases (15,16,21,23,28-33). In the present study, clustering of cases was seen in the sixth decade with a mean of 49.13 years and a median of 50 years. In contrast to the present study, Cabuk FK* et al.*, Vangangelt KMH* et al.*, Vangangelt KMH* et al.*, Downey CL* et al.,* and Roeke T* et al.* observed higher median age (15,16,21,28-30). In contrast, O’Loughling M* et al.* documented lower median age (23). Other studies had not specified the mean or median age (13,20-22). IBC was seen only in females in the present study. Downey CL* et al.* documented that IBC was seen predominantly in females in their study (29). Other studies had not specified about the gender distribution (15,16,21,23,28,30-33). Invasive breast carcinoma of no special type (IBC-NST) was the most common diagnostic entity in most of the studies including the present study. In the present study, the greatest dimension of the tumor ranged from 12mm to 100mm with a mean of 41.43mm. The greatest dimension of the tumor varied from 27mm-31mm in the study conducted by Khoury T* et al.* (32). However, other studies had not specified about the range or mean of greatest dimension of the tumor in their study (15,16,21,23,28-31,33). Grade 2 was the most common tumor grade in the present study. Cabuk FK* et al.*, Vangangelt KMH* et al.*, Downey CL* et al.*, Roeke T* et al.*, Tramm T* et al.* also documented similar observation (15, 28-31). In contrast, the most common tumor grade was grade 3 in the study conducted by Zakhartseva LM* et al.*, Vangangelt KMH* et al.*, O’Loughling M* et al.*, Khoury T* et al.* and Buisseret L* et al.* (16,21,23,32,33). In the present study, lymph node metastasis was seen in 21 cases (70%). In contrast to the present study, other studies documented a lower percentage of cases (15,16,21,28-30,33). The most common primary tumor category was pT2 in the present study. Zakhartseva LM* et al.* and Vangangelt KMH* et al.* also made similar observations (16,28). On the contrary, ypT0 was the most common primary tumor category in the study conducted by O’Loughling M* et al.* (23). Other studies had not specified the primary tumor category (15,21,29-33). The common nodal category in the present study was pN0 and pN1. The most common nodal category was pN1 in the study conducted by Zakhartseva LM* et al.* (16). O’Loughling M* et al.* documented ypN0 as the most common nodal category in their study (23). Other studies had not specified the primary nodal category in their study (15,21,28-33). Mastectomy was the only type of specimen included in the present study. O’Loughling M* et al.* conducted a study on core biopsies (23). Khoury T* et al.* conducted a study on core biopsies and full-face sections (32). However, the other studies had not specified the type of specimens in their study (15,16,21,28-31,33). 

**Comparison of Interobserver Agreement in the Evaluation of Tumor-Stroma Ratio**

In most of the studies including the present study, two pathologists participated in the evaluation of the tumor-stroma ratio. In the present study, KRAS square grid was the novel tool used for estimating the tumor-stroma ratio. Vangangelt KMH* et al.* used a circle (9 mm^2^ area) tool to estimate the tumor-stroma ratio in their study (21). Downey CL* et al.* employed a virtual graticule software in which a grid tool with a systematic random sample of 300 points was used to estimate the tumor-stroma ratio (29). In most of the studies, the protocol proposed by Mesker* et al.* was used for the estimation of TRS (16,21,30). In the present study, the protocol proposed by Mesker* et al.* was used with a minor modification to suit the requirements of the study. Since the KRAS square grid was used, a different formula was used to estimate the TSR. In most of the studies, including the present study, H&E-stained slides were used to evaluate TSR (16,21,28-30). In most of the studies, including the present study, TSR was evaluated in X100 (low power) magnification (16,21,28,30). In the present study, ten low-power fields were examined, and the average score was considered. Downey CL* et al.* evaluated only two fields (29). Other studies had not specified the exact number of fields (16,21,28,30). Most of the studies employed a two-tier system to evaluate TSR (16,21,28-30). In contrast to other studies, in the present study, a four-tier system was employed to evaluate the reproducibility of the method employed to evaluate the histopathological parameters. In most of the studies, including the present study, the Cohen Kappa coefficient was used to calculate the interobserver agreement (16,21,28-30). In the present study, agreement was 91.67% for TSR. Vangangelt KMH* et al.* documented higher agreement (28). In contrast, Roeke T* et al.* documented lower agreement (30). In the present study, the Kappa value was 0.78. Zakhartseva LM* et al.* and Vangangelt KMH* et al.* had documented higher Kappa values (16,28). In contrast, Vangangelt KMH* et al.*, Downey CL* et al.,* and Roeke T* et al.* documented lower Kappa values (21,29,30). In most of the studies, including the present study, the Kappa value suggested substantial agreement (21,29,30). The Kappa value suggested almost perfect agreement in the study conducted by Zakhartseva LM* et al.* and Vangangelt KMH* et al.* (16,28) (Table 4).

**Table 4:** Comparison of TSR parameters in various studies

Parameters	Present study	Zakhartseva LM*** et al.* (**16) (Ukraine, 2021)	Vangangelt KMH*** et al.* (**21) (The Netherlands, 2020)	Vangangelt KMH*** et al.* (**28) (The Netherlands, 2018)	Roeke T*** et al.* (**30) (The Netherlands, 2017)	Downey CL*** et al.* (**29) (United Kingdom, 2014)
Total number of the cases	30	232	1794	344	737	180
Number of the Pathologists	2	2	2	2	2	2
Tool used	RKAS square grid	-	Circle	-	-	Grid with 300 points -virtual graticule software
Protocol	Modified protocol based on Mesker* et al.*	Mesker* et al.*	Mesker* et al.*	-	Mesker* et al.*	-
Stain used	H&E	H&E	H&E	H&E	H&E	H&E
Power of the objective	10X	10X	10X	10X	10X	-
Number of fields	10	-	-	-	-	2
TSR scoring system	4 tier system	2 tier system	2 tier system	2 tier system	2 tier system	2 tier system
TSR kappa Scale	Cohen Kappa scale	Cohen Kappa scale	Cohen Kappa scale	Cohen Kappa scale	Cohen Kappa scale	-
TSR kappa value	0.78	0.84	0.61	0.85	0.68	0.70
Agreement %	91.67%	-	-	94%	85%	-
Interpretation	Substantial agreement	Almost perfect agreement	Substantial agreement	Almost perfect agreement	Substantial agreement	Substantial agreement

**Comparison of Interobserver Agreement in the Evaluation of Stromal Tumor-infiltrating Lymphocytes**

In the present study, two pathologists participated in the evaluation of the stromal tumor-infiltrating lymphocytes (sTIL). Similarly, even in the studies conducted by Khoury T* et al.* and Buisseret L* et al.*, two pathologists participated in the evaluation of the sTIL (32,33). Four pathologists participated in the study conducted by Cabuk FK* et al.* (15). Nine pathologists participated in the study conducted by Tramm T* et al.* (31). Sixteen pathologists participated in the study conducted by O’Loughling M* et al.* (23). In the present study, KRAS square grid was the novel tool used for evaluating the sTIL. The other studies had not specified any specific tool used for evaluating the sTIL (15,23,31-33). In most of the studies, sTIL was evaluated according to the international working group 2014 guidelines (15,23,31-33). In the present study, international working group 2014 guidelines with minor modifications were done to suit the requirements of the study. The number of small squares (in the square grid) predominantly (approximately >50% and/ or ≥5 lymphocytes per small square) displaying the stromal tumor-infiltrating lymphocytes were labeled, counted, and documented. In most of the studies, including the present study, H&E-stained slides were used to evaluate sTIL (15,23,31-33). O’Loughling M* et al.* and Tramm T* et al.* conducted a study on digitalized H&E slides (23,31). In most of the studies, including the present study, sTIL was evaluated in X400 (high-power) magnification (15,23,31-33). In the present study, ten high-power fields were examined, and the average score was considered. Other studies had not specified the exact number of fields evaluated (15,23,31-33). O’Loughling M* et al.* employed a two-tier system to evaluate TIL in their study (23). Tramm T* et al.* and Khoury T* et al.* employed both a two-tier system and a three-tier system to evaluate TIL (31,32). In contrast to the other study, in the present study, a four-tier system was employed to evaluate sTIL. In the present study, the Cohen Kappa coefficient as used to calculate the interobserver agreement. Cabuk FK* et al.* and Tramm T* et al.* used the Fleiss Kappa value to calculate the interobserver agreement (15,31). O’Loughling M* et al.* used the Intraclass correlation coefficient and Kappa statistics (23). Khoury T* et al.* used the Intraclass correlation coefficient (32). Buisseret L* et al.* used the Bland-Altman method, Passing-Bablok regression analysis, and concordance correlation coefficient to calculate the interobserver agreement (33). In the present study, agreement was 88.33% for sTIL. Tramm T* et al.* documented agreement in the range of 79%-95% (31). Khoury T* et al.* documented lower agreement in their study (32). In the present study, the Kappa value was 0.51. Cabuk FK* et al.*, O’Loughling M* et al.*, Khoury T* et al.* and Buisseret L* et al.* documented higher Kappa values (15,23,32,33). In contrast, Tramm T* et al.* documented a lower Kappa value (31). Kappa value suggested moderate agreement in the study conducted by O’Loughling M* et al.* and the present study (23). Kappa value suggested substantial agreement in the study conducted by Cabuk FK* et al.* (15). Kappa value suggested moderate agreement to substantial agreement in the study conducted by Khoury T* et al.* (32). Kappa value suggested fair to moderate agreement in the study conducted by Tramm T* et al.* (31). Kappa value suggested poor agreement in the study conducted by Buisseret L* et al.* (33). Hence, different studies have varied results. This variation may be due to the different statistical methods employed in different studies (Table 5).

**Table 5:** Comparison of TIL parameters in various studies

Parameters	Present study	Tramm T*** et al. ***(31) (Denmark, 2018)	Khoury T*** et al. ***(32) (New York, 2018)	O’Loughlin M*** et al. ***(23) (Ireland, 2018)	Cabuk FK*** et al. ***(15) (Turkey, 2018)	Buisseret L*** et al. ***(33) (Belgium, 2017)
Total number of the cases	30	124	100	75	121	124
Number of the Pathologists	2	9	2	16	4	2
Tool used	RKAS square grid	-				
Protocol	International working group 2014 guidelines with minor modifications	International working group 2014 guidelines	International working group 2014 guidelines	International working group 2014 guidelines	International working group 2014 guidelines	International working group 2014 guidelines
Stain used	H&E	H&E (Digitalized)	H&E	H&E (Digitalized)	H&E	H&E
Power of objective	40X	40X	40X	40X	40X	40X
Number of fields	10	-	-	-	-	-
TIL scoring system	4 tier system	2-tier and 3-tier system	2-tier and 3-tier system	2 tire system	-	-
TIL Kappa Scale	Cohen Kappa scale	Fleiss kappa value	Intraclass Correlation Coefficient, k statistics	Intraclass Correlation Coefficient	Fleiss kappa value	Bland- Altman method and Passing-Bablok regression analysis, Concordance correlation coefficient
TIL kappa value	0.51	0.36-0.44	0.53-0.65	0.595	0.78	0.69-0.71
Agreement	88.33%	79%-95%	26%-46%	-	-	-
Interpretation	Moderate agreement	Fair to moderate agreement	Moderate agreement to substantial agreement	Moderate agreement	Substantial agreement	Poor

Khoury T* et al.* opined that tumor-infiltrating lymphocytes like any morphological variables in human tissues are subjected to interobserver variability and intra-tumoral heterogeneity (32). Kos Z* et al.* enumerated various pitfalls in sTIL assessment which could be due to heterogeneity of lymphocyte distribution, technical factors, scoring wrong area or cells, and limited tumor stroma (27). 

Cabuk FK* et al.* opined that the international working group 2014 guidelines for TIL were reproducible and reliable (15). On the contrary, O’Loughling M* et al.* opined that H&E-based assessment was not sufficiently reproducible for clinical application. The author suggested the need for other methodologies to be explored (23). 

In the present study, sTIL was evaluated by KRAS square grid method. The agreement percentage was good, but the Kappa value suggested moderate agreement. This could be related to the variables like the quality of the microphotographs taken, the pathologist who captures the image, the representativeness of the field captured, experience and enthusiasm of the interpreting pathologist. Once the standardization of the above factors is taken care of, the interobserver agreement would be more acceptable. 

The Pythagorean theorem has found many applications (6). It has been used extensively in multiple disciplines, particularly the engineering field. Diagnostic availability based on Pythagoras’ theorem has been found to be a novel index for integrative evaluation of diagnostic tests (7). Pythagoras’ theorem states that the area of the square over the hypotenuse of the right-angled triangle is equal to the sum of the areas of the squares over the other two sides (6). Accordingly, if a square is inscribed in the circle, the diagonal of a square is equal to √2 the length of the side of the square. Based on the above theorem, the square grid dimension was calculated and superimposed on the microphotograph without altering the actual magnification of the photograph, and at the same time, it ensures the superimposition of a perfect square with equal dimensions of even the small squares without compromising the availability of optimal area of field required for estimating various parameters. 

It may be suggested that the concept may be extended to estimate other various parameters but not limited to histopathological parameters such as intra-tumoral tumor-infiltrating lymphocytes, mitotic figures, or immunohistochemistry marker parameters. The use of sophisticated software may be expensive and may not be a feasible option in all laboratories. The present study was done with only MS Office software which is available on all systems. No sophisticated or expensive software was used. It can be done even in low-resource laboratory settings. The concept may be utilized in the future to design an algorithm for artificial intelligence or smartphone applications at a lower cost.

Limitations of the Study

The number of cases was relatively less in comparison with other studies. This is because it was a pilot study done to ensure reproducibility of the KRAS square grid method which was utilized for the evaluation of TSR and sTIL. It was a bit time-consuming. But method adopted in the present study is more objective than the conventional visual method. For the research, ten fields were analyzed. To make it more feasible, it may be suggested that the number of fields may be reduced to five fields to minimize time consumption.

## Conclusion

The present study emphasizes the utility of the indigenously designed square grid method for the evaluation of tumor-stromal ratio (TSR) and stromal tumor-infiltrating lymphocytes (sTIL) in invasive breast carcinoma. It is a novel method that can be considered an example of the application of Pythagoras’ theorem in pathology used for the purpose of analysis of microscopic images. It can be performed even in low-resource laboratory settings. It may be suggested that the concept may be extended to estimate other various parameters but not limited to histopathological parameters or immunohisto-chemistry parameters. The concept may be utilized in the future to design an algorithm for artificial intelligence or smartphone applications at a lower cost.

## Funding

The authors received no financial support for the research, authorship, and/or publication of this article.

## Conflict of Interest

The authors declared no conflict of interest.
